# Development of a deep learning model for automated diagnosis of neuromuscular diseases using ultrasound imaging

**DOI:** 10.3389/fneur.2025.1640428

**Published:** 2025-09-10

**Authors:** Jingyi Xie, Zhenying Zhang

**Affiliations:** ^1^School of Physical Education, Central China Normal University, Wuhan, China; ^2^Department of Physical Education and Military Training, Zhejiang Agriculture and Forestry University, Hangzhou, China

**Keywords:** neuromuscular disease, ultrasound image classification, deep learning, lightweight neural network, model interpretability

## Abstract

**Background:**

Neuromuscular diseases (NMDs) pose significant diagnostic challenges due to their heterogeneous clinical manifestations and the limitations of traditional diagnostic tools. While musculoskeletal ultrasound has become a promising non-invasive modality for evaluating muscle pathology, its diagnostic accuracy remains heavily dependent on the operator’s expertise. To address this, we propose a lightweight and interpretable deep learning model to enable automated classification of ultrasound images in NMD screening.

**Method:**

We developed a novel model, termed NMD-AssistNet, which integrates GhostNet as the backbone with CBAM attention modules and depthwise separable convolutions to enhance both efficiency and discriminative capacity. The model was trained and evaluated on a public dataset containing 3,917 annotated ultrasound images of various muscle groups. Mixup augmentation, label smoothing, and SWALR learning rate scheduling were applied to improve generalizability. Performance was benchmarked against CSPNet, EfficientNet, GhostNet, HRNet, and Vision Transformer.

**Results:**

NMD-AssistNet achieved the highest performance among the evaluated models, reaching a classification accuracy of 0.9502 and an area under the curve (AUC) of 0.9776. Grad-CAM visualizations revealed that the model effectively focused on clinically relevant muscle regions, highlighting its potential interpretability.

**Conclusion:**

NMD-AssistNet demonstrates strong diagnostic capability, computational efficiency, and model interpretability and offers a promising solution for real-time, automated NMD screening. This framework has the potential to be deployed in portable ultrasound systems or edge AI devices to assist clinicians in both hospital and community healthcare settings.

## 1 Introduction

Neuromuscular diseases (NMDs) are a class of complex diseases that originate from the anterior horn of the spinal cord, peripheral nerves, neuromuscular junctions, or skeletal muscles. Their clinical manifestations include a variety of symptoms such as muscle atrophy, decreased muscle strength, impaired motor function, and even respiratory insufficiency (1). Different types of NMDs vary significantly in disease progression, prognosis, and treatment response. Clinical intervention strategies are also highly dependent on the accurate identification of disease types and their stages (2). While a detailed neurological examination is the cornerstone of diagnosis and can often differentiate the level of the lesion, the diagnostic process can still be challenging and protracted. This is particularly true in the early stages of the disease, where symptoms may be subtle or overlap with other neurological conditions, necessitating objective diagnostic aids to supplement clinical assessment (3).

Ultrasound imaging, as a non-invasive, dynamic and repeatable imaging technology, has been widely used in the clinical evaluation of muscle diseases in recent years. Compared with traditional methods such as electromyography (EMG) or muscle biopsy, ultrasound can not only reflect changes in muscle structure in real time, such as changes in muscle thickness, echo intensity and texture pattern, but can also be used to dynamically observe the muscle’s behavior during movement, thereby assisting in the assessment of disease activity and treatment effect (4). However, this imaging technology is highly dependent on the operator’s technical level and clinical experience, and different doctors may have different interpretations of the same image. In addition, the performance of muscle tissue in ultrasound images is often complex and variable due to disease type, individual differences and different scanning angles. Factors such as blurred lesion boundaries and overlapping tissue echoes may interfere with diagnostic judgment (5).

To address the shortcomings of traditional image interpretation methods, the application of artificial intelligence (AI), especially deep learning, in the field of medical image processing has gradually deepened in recent years. Recent comprehensive reviews have highlighted that AI, and particularly deep learning, holds significant promise for transforming diagnostic workflows in neuromuscular medicine (6). A convolutional neural network (CNN) is a specialized deep learning architecture for processing grid-like data, such as an image. Its key advantage is the ability to automatically learn relevant features from the input data through the use of convolutional filters. In the imaging diagnosis of neuromuscular diseases, CNNs are widely used for automatic feature extraction and image classification tasks. Their advantage is that they can autonomously learn and extract multi-level texture information from complex image data (7). At the same time, transfer learning strategies have been introduced to overcome the limitations of small sample data sets on model training, and have shown good adaptability under the realistic conditions of scarce medical images (8). The multi-task learning framework improves the spatial sensitivity and discrimination of the model to the lesion area by simultaneously optimizing target tasks such as classification and segmentation, thereby improving the overall diagnostic performance (9).

Although the above-mentioned technological progress is encouraging, there are still many technical bottlenecks that limit its clinical promotion. First, the performance of existing models in real ultrasound scenes facing high noise, low contrast and complex backgrounds is still unstable, and it is prone to misjudgment and missed diagnosis (10). More importantly, the vast majority of studies are based on small, single-center datasets, and the generalization ability and robustness of the model have not been fully verified in a multi-institutional and heterogeneous equipment environment, limiting its translation from scientific research to clinical practice. Therefore, developing a lightweight, high-performance neural network architecture with good generalization ability to improve the efficiency and stability of auxiliary diagnosis of neuromuscular diseases in ultrasound images has become an important direction of current medical artificial intelligence research.

**Figure 1:**
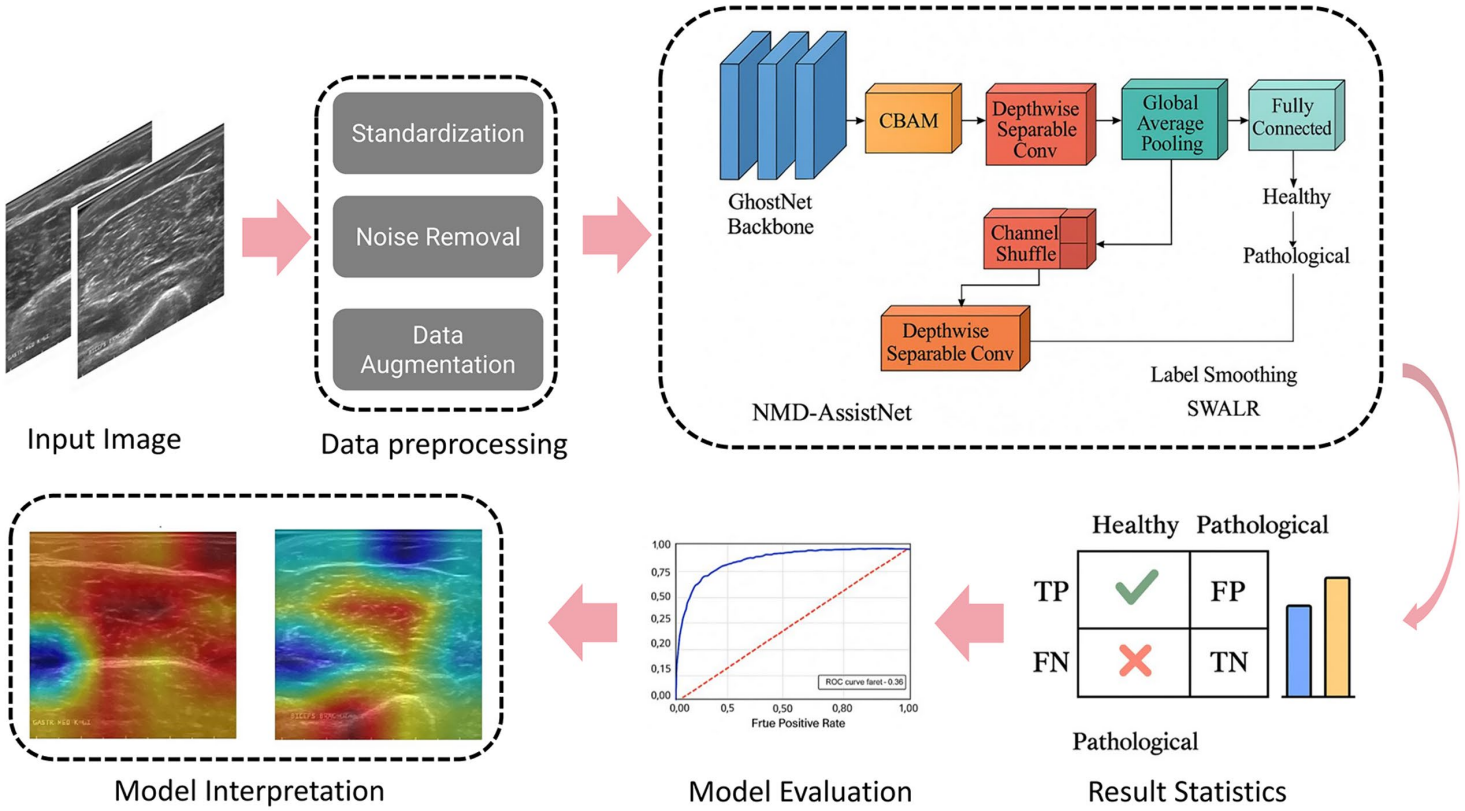
The overall technical route of this study.

To address the aforementioned challenges, this paper proposes NMD-AssistNet, a novel lightweight deep learning model for the efficient and automated classification of neuromuscular diseases from ultrasound images. Based on the GhostNet architecture (11), this model introduces the CBAM (Convolutional Block Attention Module) that integrates channel attention and spatial attention to enhance the feature focusing ability of key lesion areas. At the same time, it combines depthwise separable convolution and a channel shuffling mechanism to significantly reduce the number of model parameters and computational complexity, thereby adapting to the characteristics of complex texture details and blurred boundaries in ultrasound images. In addition, in order to improve the generalization ability and training stability of the model, we also introduced the Mixup data enhancement strategy, label smoothing regularization, and SWALR (Stochastic Weight Averaging with Learning Rate Scheduling). The experimental part is based on a real clinical ultrasound image dataset, and systematically compares its performance with multiple mainstream models on multiple classification performance indicators, and analyzes its diagnostic accuracy and clinical interpretability through attention heat maps (Figure 1).

## 2 Methods

### 2.1 Data collection

This study utilized a public ultrasound image dataset from the Mendeley data repository (DOI: 10.17632/3jykz7wz8d.1), originally collected in the Arnhem-Nijmegen region. The dataset is governed by the Creative Commons Attribution 4.0 (CC BY 4.0) license, which explicitly permits data reuse for any research purpose with proper citation, thus no separate institutional review board approval was required for our secondary analysis (12). The dataset contains data from a total of 1,283 subjects (average age 50 ± 21 years, 729 males), including 3,917 cross-sectional ultrasound images, covering three common skeletal muscle groups: biceps brachii, tibialis anterior, and medial gastrocnemius. All images were manually annotated by experienced clinical ultrasound experts to ensure the accuracy of anatomical structure identification and classification labels (13). To classify muscle images into healthy and pathological categories, the original research team used a z-score method based on grayscale values. The z-score was calculated using linear regression models derived from a healthy population, incorporating variables such as age, gender, and BMI. An image was labeled as “abnormal” if its grayscale value z-score was greater than 2, and “normal” otherwise. This classification resulted in the following distribution per muscle group: biceps brachii (287 normal, 158 abnormal subjects), medial gastrocnemius (266 normal, 110 abnormal subjects), and tibialis anterior (296 normal, 166 abnormal subjects). In total, the dataset comprises images from 849 normal and 434 abnormal subjects (Table 1 and Supplementary Table S1).

**Table 1:** Description of the public neuromuscular ultrasound dataset.

Characteristic	Details
Total subjects	1,283
Subjects with normal images	849
Subjects with abnormal images	434
Demographics	729 males; average age: 50 ± 21 years
Total images	3,917 (approx. three to four images per subject per muscle)
Muscles included	Biceps brachii, tibialis anterior, medial gastrocnemius
Pathology type	Neuromuscular disorders (e.g., myopathy, neuropathy)
Image specifications	B-mode, PNG format
Data source	Mendeley data (doi: 10.17632/3jykz7wz8d.1)

### 2.2 Data preprocessing

Before model training, we performed a systematic data preprocessing pipeline on all ultrasound images to ensure input data quality, structural consistency, and to promote model training stability and convergence. This pipeline included size unification, noise suppression, and image enhancement. First, we performed image standardization and cleaning. To meet the input requirements of the deep learning model, all images were uniformly resized to 224 × 224 pixels. Given that ultrasound images are inherently affected by speckle noise, we employed a median filter with a 5 × 5 kernel (14). This method was chosen for its effectiveness in suppressing salt-and-pepper-like noise while preserving critical muscle boundary details better than linear filters like Gaussian blur. Subsequently, to address the issue of variable contrast across different images, which can arise from different equipment settings or patient tissues, we applied Contrast Limited Adaptive Histogram Equalization (CLAHE) instead of global histogram equalization. CLAHE enhances local contrast without over-amplifying noise in relatively uniform regions, making it particularly suitable for medical image analysis.

Considering the high heterogeneity of clinical ultrasound images, we implemented a comprehensive data augmentation strategy to enhance the model’s robustness and mitigate overfitting (15). For the training set, we applied several transformations with specified probabilities: resized cropping (to 224 × 224), horizontal flipping (*p* = 0.5), and perturbations in brightness, contrast, and saturation (with a jitter factor of 0.2). On this basis, we further introduced the Mixup data augmentation strategy, with the mixing coefficient *λ* drawn from a Beta distribution (*α* = 0.2, *β* = 0.2). This method generates virtual training samples by linearly combining two images and their corresponding labels, which has been shown to improve model generalization and performance on imbalanced datasets (15).

For label processing, we encoded the original diagnostic labels (“normal” and “abnormal”) into a binary format: 0 for normal and 1 for pathological. This was based on the z-score grading results, and a mapping dictionary was constructed to ensure consistent annotation during training and validation. Additionally, to meet the input requirements of NMD-AssistNet, the images were converted to tensors and normalized at the channel level using PyTorch’s built-in Normalize function. The mean and standard deviation were set to the standard values used by the ImageNet pre-trained model ([0.485, 0.456, 0.406] and [0.229, 0.224, 0.225]), respectively, to facilitate the application of transfer learning strategies (16). To systematically validate the contribution of each component within this preprocessing pipeline, we designed an ablation study. This study involved incrementally adding each processing step to a baseline model to quantify its specific impact on performance.

### 2.3 Region of interest segmentation

Although the main task of this study focuses on the classification of ultrasound images of neuromuscular diseases, due to the fact that muscle tissue in ultrasound images often has features such as blurred boundaries, uneven grayscale distribution, and complex background structures, direct classification training on the entire image may cause the model’s attention to diverge and interfere with the accuracy of feature extraction. Therefore, before the image is input into the model, we introduced the muscle contour information based on expert annotation to extract the region of interest (ROI) to weaken irrelevant background interference and enhance the model’s ability to focus on key structures. Each image in the dataset is accompanied by a muscle cross-sectional area (CSA) contour mask drawn by experts. These masks were originally used for grayscale analysis and muscle volume estimation. In this study, we converted it into the cropped area required for the classification task, extracted the minimum rectangular area containing the muscle body by calling the contour boundary coordinates, and performed appropriate upper, lower, left, and right edge expansion on this basis to retain the surrounding important structural information. The processed ROI images were uniformly adjusted to the standard input size and replaced the original full images in the subsequent model training process. This strategy effectively reduces the risk of model learning background noise while retaining the core muscle tissue features, and improves the focus and discriminability of feature extraction. It is worth pointing out that this study did not train a pixel-level segmentation model separately, but made full use of the existing manually annotated segmentation mask as a guide to construct better classification input samples through ROI cropping. The reliability of these masks is critical. The ground-truth segmentation was performed by two experienced clinicians (a physiotherapist and a technical physician). They first annotated images independently, then resolved discrepancies via consensus discussion (12).

### 2.4 Diagnostic model construction

#### 2.4.1 NMD-AssistNet

In order to achieve efficient and accurate identification of neuromuscular diseases, this paper designs a lightweight deep learning model tailored for ultrasound image diagnosis tasks, named NMD-AssistNet (Neuromuscular Disease Assistive Network). The model uses an improved GhostNet as the backbone architecture, combined with an attention mechanism and a lightweight convolution strategy, to significantly reduce the number of model parameters and computational overhead while ensuring diagnostic performance, thereby meeting the comprehensive requirements of medical image analysis for efficiency, generalization ability, and clinical deployability.

NMD-AssistNet consists of three key modules: the basic backbone adopts the GhostNet framework to obtain efficient sparse feature representation; the feature enhancement part introduces the channel and space dual attention mechanism—CBAM to enhance the model’s ability to pay attention to the texture and structure of the lesion area (17); at the same time, the model embeds depthwise separable convolution and channel shuffle operations in multiple convolutional layers to further compress parameters, improve operation speed, and enhance the fusion and flow of multi-scale features (12). The final output layer of the model uses global average pooling combined with a fully connected layer to map to a binary classification label space to achieve automatic judgment of “healthy” and “pathological” images. We introduced label smoothing technology at the loss function level to effectively alleviate the risk of overfitting during training. At the same time, in terms of training scheduling strategy, the SWALR method is used, combined with periodic learning rate adjustment and model weight averaging to improve the optimization convergence speed and obtain more stable model performance (18).

#### 2.4.2 Model comparison

In order to systematically evaluate the performance advantages of NMD-AssistNet, this study selected five representative deep learning models for comparison, including CSPNet (12), EfficientNet (12), GhostNet (19), HRNet (20), and Vision Transformer (ViT) (21). These models cover different technical paths such as lightweight design, multi-scale modeling and global attention mechanism, representing the current mainstream modeling strategies for medical image classification. CSPNet and GhostNet emphasize high efficiency, but have deficiencies in feature expression and lesion perception; although EfficientNet has strong performance, its large network size limits clinical deployment; HRNet maintains high-resolution features and has ideal boundary preservation effects, but has high computational complexity; ViT has global modeling capabilities and is suitable for large-scale natural image classification, but is prone to overfitting in small and medium sample medical scenarios.

### 2.5 Experimental setup

To ensure a rigorous and clinically meaningful evaluation, the dataset was partitioned at the subject level to prevent data leakage, where all images from a single individual were exclusively allocated to either the training or the validation set. We employed a stratified random sampling strategy based on the diagnostic label (normal vs. abnormal) to maintain a consistent class distribution across the sets. From the total of 1,283 subjects, 898 subjects (70%) were allocated to the training set, and the remaining 385 subjects (30%) were assigned to the validation set. This subject-level split resulted in the following data distribution: (1) Training set: Comprised 898 subjects (595 normal, 303 abnormal), totaling approximately 2,742 images. (2) Validation set: Comprised 385 subjects, totaling 783 images. The model was trained on an Ubuntu 20.04 platform configured with an Intel i9-12900K CPU, 128GB RAM, and an NVIDIA RTX 3090 GPU (24GB). The development environment included PyTorch 1.13.1, CUDA 11.7, and cuDNN 8.4.1 (22). The training used the Adam optimizer (initial learning rate 0.001, batch size 32, weight decay 1e-5), and combined with the SWALR strategy to dynamically adjust the learning rate to improve convergence efficiency. The total number of training epoch was set to 100, and the early stopping mechanism was enabled to prevent overfitting. All comparison models (CSPNet, EfficientNet, GhostNet, HRNet, and ViT) were uniformly trained on the same data and environment to ensure experimental fairness.

### 2.6 Model evaluation

We evaluated model performance using several quantitative metrics, including accuracy, precision, recall, F1-score, and the area under the curve (AUC). Accuracy measures the proportion of correct classifications and is the basic indicator for evaluating overall performance. Precision (also known as positive predictive value, PPV) is used to evaluate the proportion of the model’s predictions of the “pathology” category that are actually pathological, reflecting its ability to control false positives. Recall measures the model’s ability to identify pathological samples and reflects the risk of missed diagnosis. The F1-score, as the harmonic mean of precision and recall, is suitable for scenarios with uneven sample distribution. Finally, the AUC value reflects the stability and discrimination ability of the model at different decision thresholds and is an important basis for evaluating the generalization performance of the model (23). In addition to these quantitative metrics, we qualitatively assessed the model’s interpretability using Gradient-weighted Class Activation Mapping (Grad-CAM), a technique that generates a visual heatmap to identify the image regions most influential for the model’s classification decision. In these visualizations, the heatmap is used for a qualitative assessment of the model’s focus. Warmer colors (e.g., red) indicate regions that were more influential in the model’s classification decision, while cooler colors (e.g., blue) represent areas of lesser importance. This approach helps to interpret where the model is looking, rather than quantifying the precise importance of each pixel.

## 3 Results

### 3.1 NMD-AssistNet performance

#### 3.1.1 Overall performance

**Figure 2:**
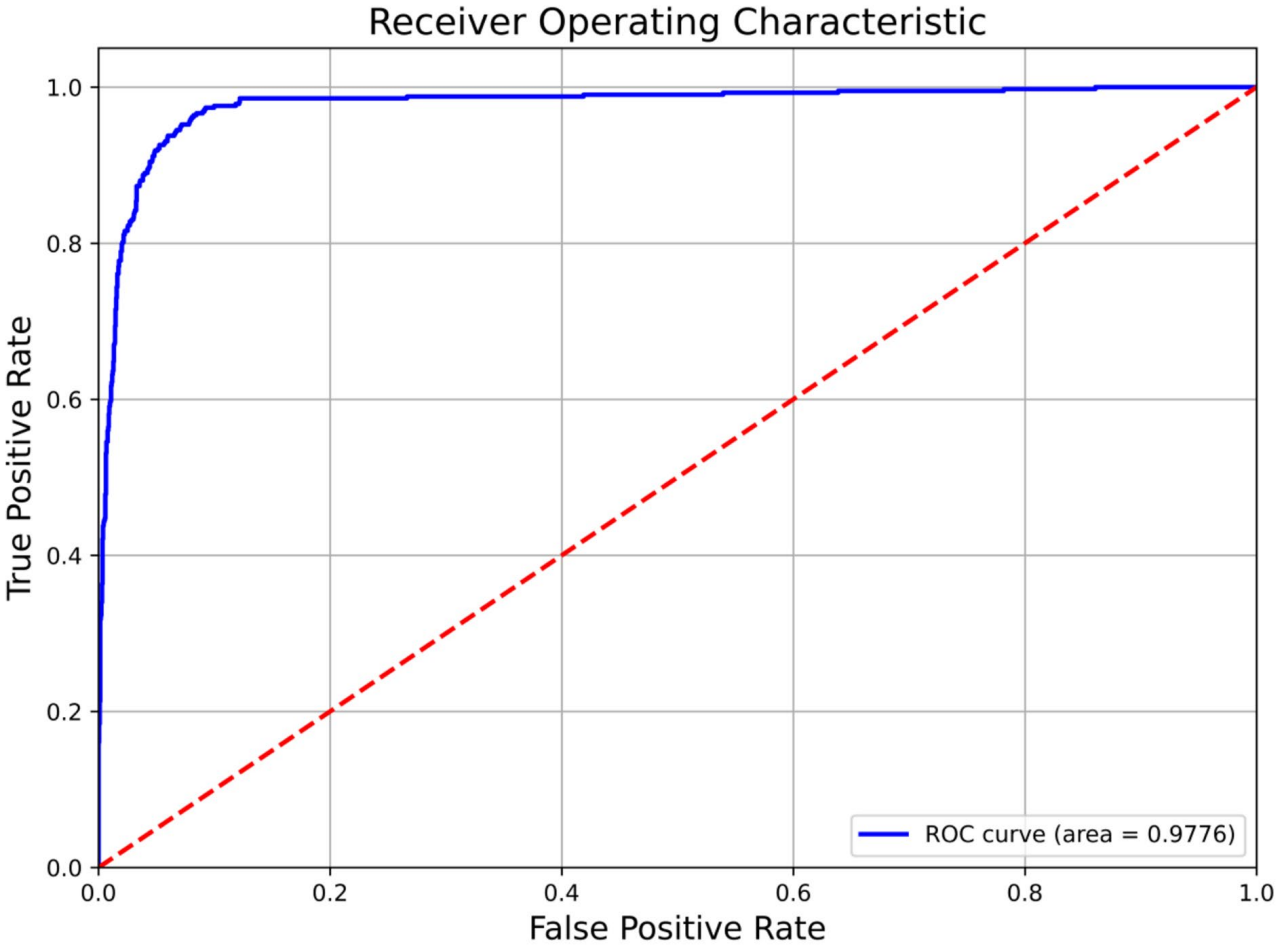
ROC curve and corresponding AUC value of the NMD-AssistNet model on the validation set.

NMD-AssistNet achieved high performance across all evaluated metrics (Table 2). The model correctly classified 738 out of 783 total samples, with the full confusion matrix detailed in Table 2. Figure 2 shows the receiver operating characteristic (ROC) curve for NMD-AssistNet, which is positioned close to the upper-left corner with an AUC value of 0.9776.

**Table 2:** Detailed performance metrics and confusion matrix.

Metric	Value	Confusion matrix	Value
Accuracy	0.9502	True positives (TP)	240
Sensitivity (recall)	0.9177	True negatives (TN)	498
Specificity	0.9540	False positives (FP)	24
Precision (PPV)	0.9085	False negatives (FN)	21
Negative predictive value (NPV)	0.9595	AUC	0.977
F1-score	0.9130	Trainable parameters	2,395,768

#### 3.1.2 Ablation study on preprocessing methods

An ablation study was conducted to validate the effectiveness of each component in the data preprocessing pipeline. As detailed in Table 3, each added step incrementally improved model performance. The inclusion of standard data augmentation (Exp. D) yielded the most substantial increase in F1-score, while the complete pipeline with Mixup (Exp. E) achieved the highest scores across all metrics.

**Table 3:** Ablation study on the contribution of each data preprocessing component to the model’s performance.

Exp. no.	Preprocessing methods	Accuracy	Precision	Recall	F1-score	AUC
A	Baseline (resizing and normalization only)	85.20%	84.50%	85.80%	85.10%	0.915
B	+Median filter	86.00%	85.20%	86.70%	85.90%	0.923
C	+Median filter + CLAHE	87.10%	86.50%	87.70%	87.10%	0.935
D	+Median filter + CLAHE + standard augmentation	91.60%	90.50%	91.50%	91.00%	0.97
E	Full pipeline (+median filter + CLAHE + standard augmentation + mixup)	92.70%	91.90%	92.50%	91.30%	0.977

### 3.2 Comparison of model results

**Figure 3:**
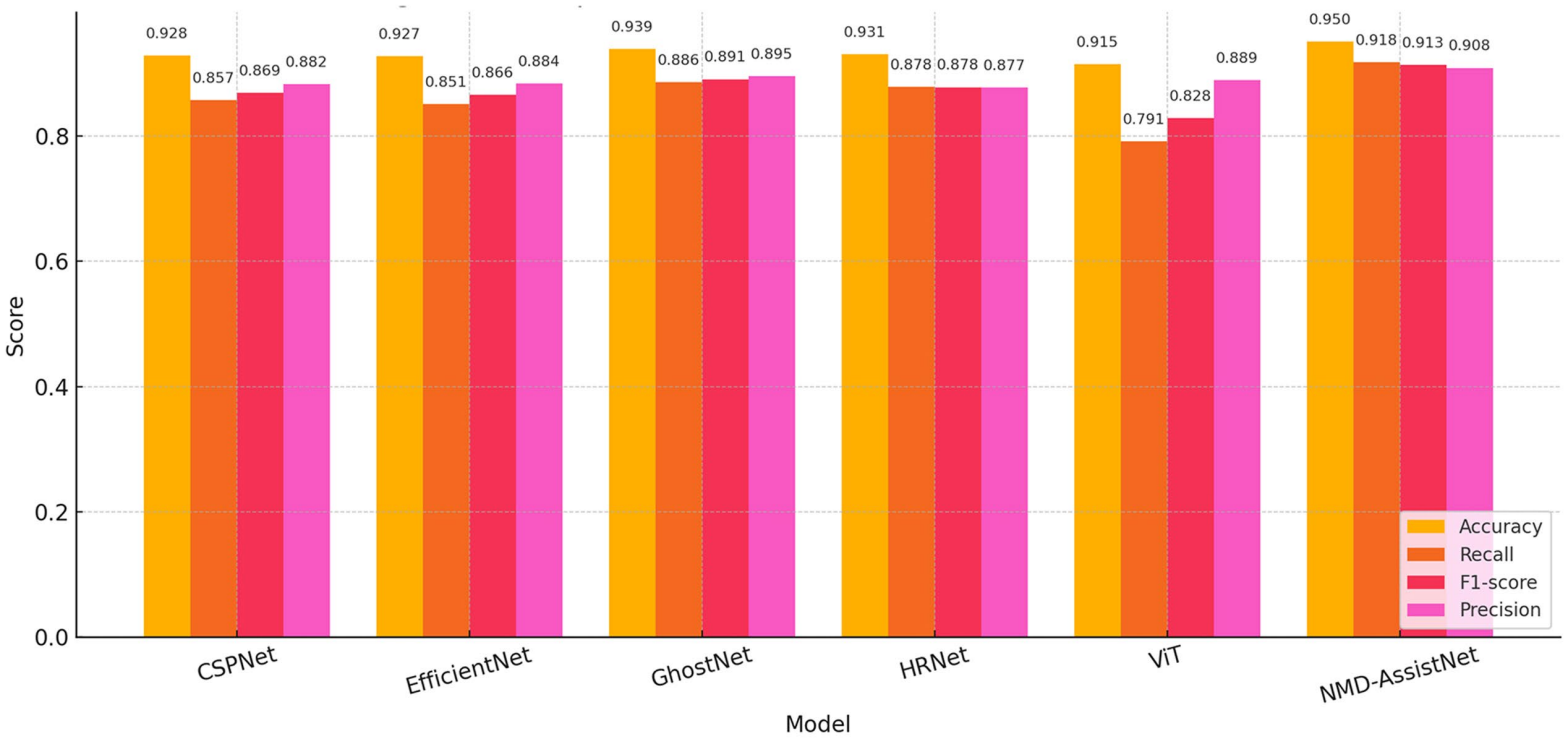
Performance comparison of classification metrics. Bar chart comparing accuracy, recall, precision, and F1-score across six models.

**Figure 4:**
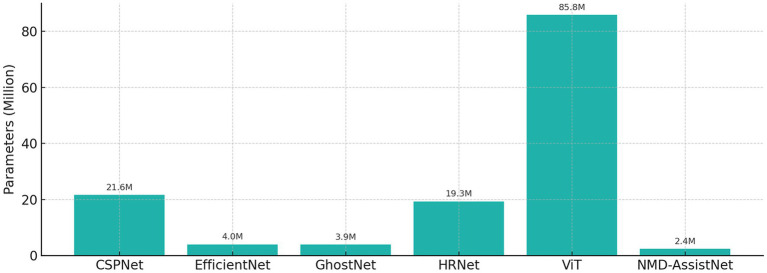
Model size comparison by trainable parameters, showing the number of trainable parameters for each model. NMD-AssistNet has the smallest model size (2.4 M) while maintaining top performance, demonstrating strong potential for edge deployment.

**Figure 5:**
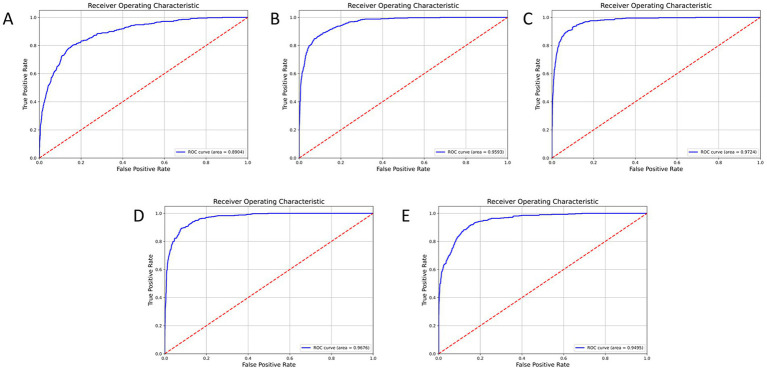
ROC curve and AUC comparison among models on the validation set. NMD-AssistNet achieved the highest AUC (0.9776), outperforming CSPNet (**A**, 0.8904), EfficientNet (**B**, 0.9593), GhostNet (**C**, 0.9724), HRNet (**D**, 0.9676), and ViT (**E**, 0.9495).

To evaluate its performance relative to other models, NMD-AssistNet was compared with five mainstream architectures under identical conditions. As shown in Figure 3, NMD-AssistNet achieved the highest values across all four metrics (accuracy, recall, precision, and F1-score) when compared to CSPNet, EfficientNet, GhostNet, HRNet, and ViT. Figure 4 illustrates that the parameter count of NMD-AssistNet (2.4 M) is substantially lower than that of the other models. The ROC analysis further shows that the AUC for NMD-AssistNet (0.979) was higher than all other tested models (Figure 5).

#### 3.2.1 Grad-CAM display and analysis of the model

**Figure 6:**
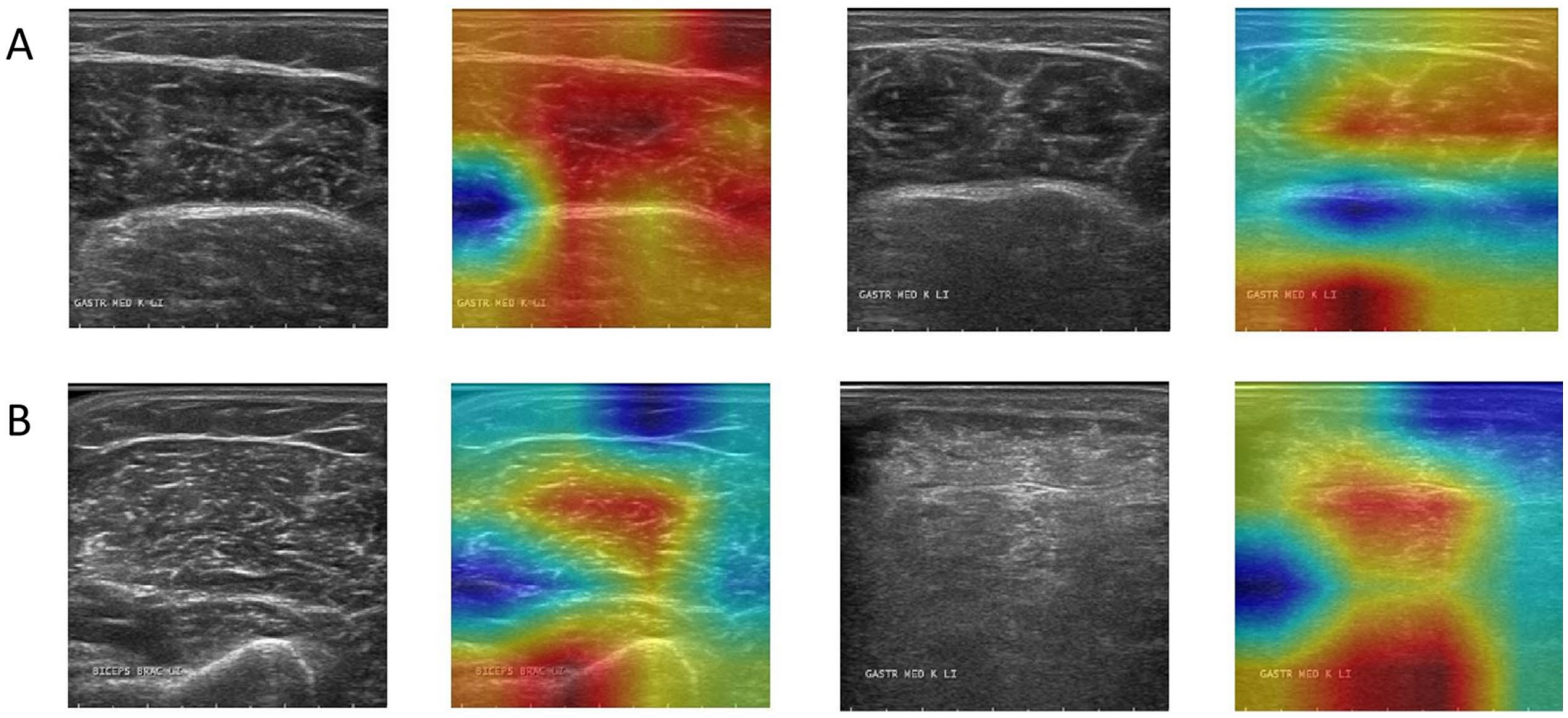
Grad-CAM activation heatmap visualization, where **(A)** represents healthy samples and **(B)** represents abnormal samples.

Grad-CAM was used to visualize the model’s focus areas on typical healthy and pathological muscle images (Figure 6). For healthy samples (Figure 6A), the high-response areas in the heat map concentrated on the transverse stripe-like texture corresponding to neatly arranged muscle fibers. For the pathological sample (Figure 6B), the high-response areas focused on regions with disordered echoes, blurred edges, or focal hypoechoic areas. These visualized regions correspond to locations of known pathological features.

## 4 Discussion

This study developed NMD-AssistNet, an efficient and lightweight deep learning model designed to meet the clinical need for auxiliary diagnosis of neuromuscular diseases by automatically classifying muscle tissue in ultrasound images. The model demonstrated strong performance, achieving an accuracy of 0.9502 and an AUC of 0.9776. This success is attributed to its hybrid architecture. By integrating the lightweight GhostNet framework with a CBAM attention mechanism, depthwise separable convolution, and channel shuffle strategies, NMD-AssistNet effectively focuses on key pathological features while maintaining a remarkably low parameter count of 2.4 M, which is significantly lower than larger models like ViT and HRNet. Furthermore, advanced training strategies, including Mixup augmentation, label smoothing, and SWALR, were employed to enhance the model’s generalization and stability, proving the effectiveness of our design.

A critical analysis of the Grad-CAM visualizations, however, warrants a more nuanced discussion. As observed, the heatmaps occasionally highlight the boundary between the ROI and the masked background. This phenomenon can be attributed to the nature of Grad-CAM interacting with the pre-segmented dataset used in this study (24). The artificial, sharp edge resulting from the expert-provided segmentation masks creates a high-gradient region, which can become a prominent focus for the visualization technique (25). While this may suggest the model is learning from edge artifacts, it is crucial to interpret this alongside the model’s high accuracy. The strong performance indicates that the model successfully learns discriminative features from within the muscle tissue itself, as these are essential for distinguishing between healthy and pathological patterns. The boundary highlighting is more likely a byproduct of the visualization technique interacting with pre-processed data, rather than the sole basis for the model’s decision. This observation underscores a key challenge in applying post-hoc interpretability methods to pre-segmented images and reinforces the importance of our future research direction (26). Developing an end-to-end model that operates on unsegmented, raw ultrasound images would not only streamline the clinical workflow but also yield more authentic interpretability results, ensuring the model’s focus is entirely on natural anatomical and pathological features.

It is crucial to contextualize the role of NMD-AssistNet within the broader clinical diagnostic pathway. Neuromuscular diseases are characterized by hidden onset, diverse symptoms, and high misdiagnosis rates (27). Our model’s function is to provide a rapid, objective classification of a single muscle’s ultrasound image as ‘pathological’ or ‘healthy’; it does not, by itself, identify the specific type of NMD. The definitive diagnosis of NMDs relies on a comprehensive evaluation, where identifying the specific pattern of affected and spared muscle groups is paramount (28). For instance, patterns of selective muscle involvement are critical clues that help clinicians narrow the differential diagnosis among various muscular dystrophies or myopathies before proceeding to genetic testing or muscle biopsy.

Therefore, the primary clinical impact of NMD-AssistNet is its potential as a powerful adjunctive screening tool, rather than a standalone diagnostic solution. First, it can provide an objective, quantitative assessment of muscle echogenicity, reducing the inter-observer variability common in ultrasound interpretation and helping less experienced clinicians make more reliable initial judgments. Second, in a clinical setting, a rapid “abnormal” finding from NMD-AssistNet can help prioritize patients for further, more invasive and costly investigations like electromyography (EMG) or genetic panels. Finally, due to its non-invasive nature, the tool could be used longitudinally to objectively monitor disease progression or treatment response. The model’s low computational overhead makes it highly suitable for deployment on portable ultrasound equipment or edge computing devices, bringing intelligent diagnostic support to primary care facilities and community screening scenarios.

Although this study has achieved good results in model design and performance, there are still several limitations that cannot be ignored in the process of clinical transformation and practical application. Firstly, a primary limitation is the model’s reliance on pre-segmented ROIs. This approach hinders clinical efficiency. Furthermore, it can create visual artifacts at the ROI boundaries in Grad-CAM maps, complicating interpretability. Therefore, future work will focus on developing an end-to-end model to analyze unsegmented images, which would simultaneously streamline the diagnostic process and provide more authentic visual explanations. Secondly, the use of single-center data is a key limitation, necessitating external validation to ensure the model can generalize across different equipment and patient populations. To this end, validating our model on public datasets is our crucial next step to prove its potential as a clinically viable tool. Thirdly, the current model only performs a binary classification (healthy vs. pathological) and has not yet been refined to differentiate specific subtypes of neuromuscular diseases, such as muscular dystrophy or myasthenia gravis. This limits the model’s immediate applicability for refined diagnosis and individualized treatment planning. Finally, our dataset was partitioned into only training and test sets, without a separate, dedicated validation set for hyperparameter tuning. While we mitigated this by performing model selection using cross-validation within the training data, future work could benefit from a three-way split (training, validation, and test). This would provide an even more robust framework for model development and evaluation, further ensuring the generalizability of the results.

## 5 Conclusion

This study introduces NMD-AssistNet, a lightweight deep learning model for the automated classification of neuromuscular diseases from ultrasound images. The primary significance of our work lies in achieving high diagnostic accuracy while maintaining exceptional computational efficiency. This balance is critical for clinical translation, as it enables the model’s potential deployment on portable ultrasound systems or edge devices, thereby facilitating point-of-care screening. Furthermore, our interpretability analysis using Grad-CAM confirms that the model’s decisions are based on clinically relevant tissue features, a crucial step for building trust and acceptance among clinicians. While the results are promising, we acknowledge that future work must validate the model’s generalizability on larger, multi-center datasets. The next logical step is to evolve this framework from a binary classifier into a multi-class system capable of differentiating specific disease types. In summary, NMD-AssistNet provides a viable technical approach for developing intelligent, efficient, and trustworthy AI-powered tools to augment the clinical workflow for neuromuscular disease diagnosis.

## Data Availability

The original contributions presented in the study are included in the article/Supplementary material, further inquiries can be directed to the corresponding author.
