# Supplementary material for: Development of a deep learning model for automated diagnosis of neuromuscular diseases using ultrasound imaging
Source: Front Neurol. 2025 Sep 10;16:1640428. doi: 10.3389/fneur.2025.1640428 (PMC12457126; doi:10.3389/fneur.2025.1640428)
Supplement: Supplementary file 1 [file Data_Sheet_1.docx]

Table S1 Representative examples of NMD-AssistNet's class activation maps on diverse healthy muscle images

| 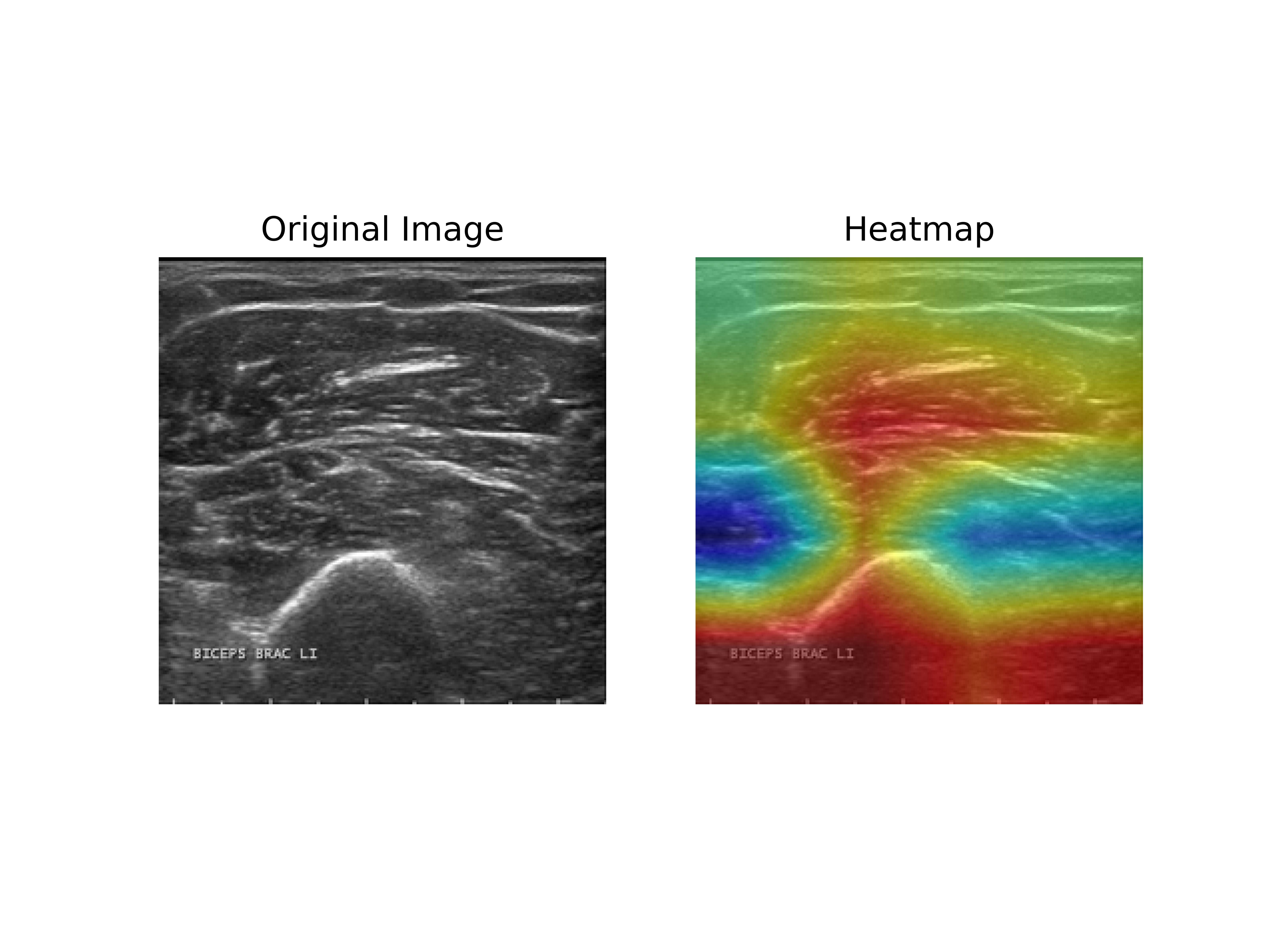 | ****A classic example of a healthy Biceps Brachii (BB).**** It shows clear muscle texture and well-defined borders. The heatmap accurately focuses on the muscle parenchyma. |
| --- | --- |
| 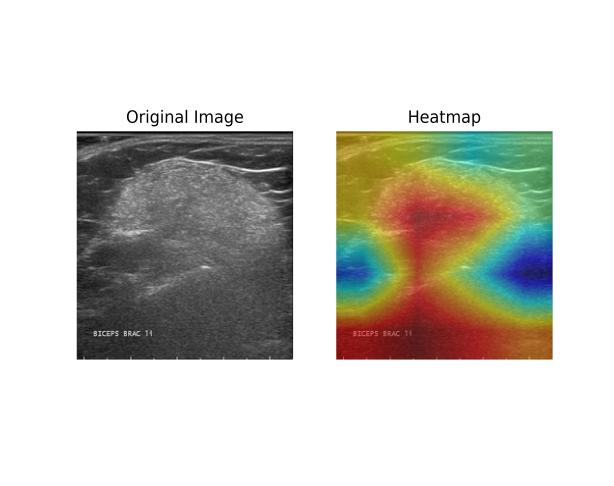 | ****A representative case of a pathological BB.**** The image shows increased echogenicity and disorganized texture. The heatmap's diffuse pattern, different from the healthy sample, highlights the model's ability to identify pathological features. |
| 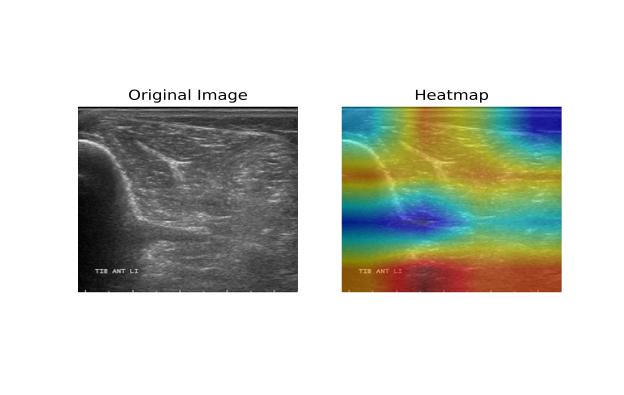 | ****A healthy Tibialis Anterior (TA) muscle,**** exhibiting a clear pennate structure. The heatmap's activation aligns precisely with the direction of the muscle fibers. |
| 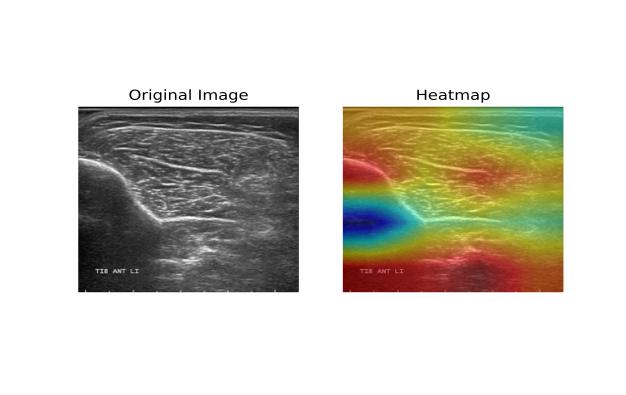 | ****A pathological TA with muscle atrophy and heterogeneous echotexture.**** The heatmap's activation area and pattern starkly contrast with its healthy counterpart. |
| 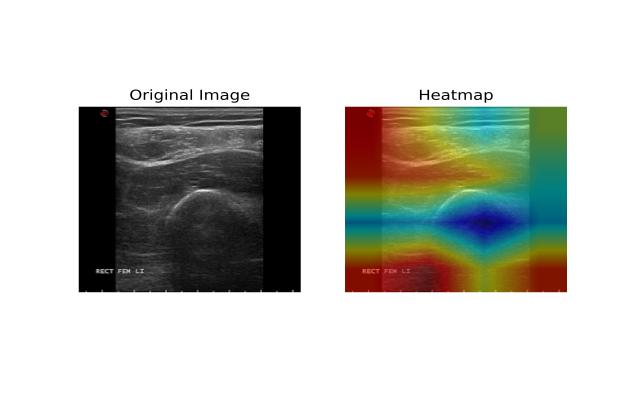 | ****A healthy Rectus Femoris (RF) muscle.**** Including a third muscle type demonstrates the model's generalizability. The heatmap is focused and accurate. |
| 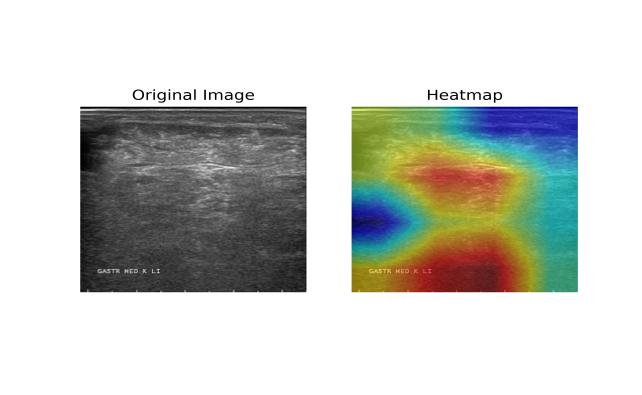 | ****A pathological Gastrocnemius (GM) muscle,**** showing abnormalities in a fourth muscle type. The image exhibits signs of atrophy and fatty infiltration, which are correctly captured by the heatmap. |
